# Is it Attention Deficit Hyperactivity Disorder (ADHD) or Stimulant use disorder ? How is ADHD diagnosed?

**DOI:** 10.1192/j.eurpsy.2022.1516

**Published:** 2022-09-01

**Authors:** N. Sud

**Affiliations:** Bamburgh Clinic, St Nicholas Hospital, Newcastle upon Tyne, United Kingdom

**Keywords:** ADHD, stimulant, Child and adolescent psychiatry, primary care

## Abstract

**Introduction:**

From clinical experience, majority of patients in adult forensic services who have childhood diagnosis of ADHD self-report onset of substance misuse around the age of 12.

**Objectives:**

Aim of the study was to explore if routine screening of childhood substance use is considered by ADHD diagnostic services.

**Methods:**

PsycINFO and Embase databases were searched with the keywords: Attention Deficit Hyperactivity Disorder, ADHD, primary care/general practice, family physicians ,paediatrics, and children/adolescents, child and adolescent psychiatry, diagnostic assessments, substance / drug use, prescription drugs and drug screening.

**Results:**

24 articles were retrieved for age groups 12 to 17 years. Studies identified substance misuse as highly comorbid with ADHD but more so in conduct disorder. Studies identified diversion risk in adolescents.

**Conclusions:**

Both ADHD and amphetamine misuse disorders are Axis 1 disorders (Baldwin 2009). Literature links substance misuse in ADHD to conduct disorder. There needs to be research into the diagnostic overlap between conduct disorder and ADHD and how this fits into the trauma model of adult offender treatment pathways. Treatment pathways for ADHD or conduct disorder and childhood onset substance misuse disorder are not clear both in primary or secondary care. Literature appears to put emphasis on early diagnosis and prescription stimulant treatment outside the social and psychological context and cites outcomes of the short term studies as reason for continued prescriptions in adolescence and beyond. There is need for studies exploring perspectives and trajectories of amphetamine use in adults who were diagnosed with ADHD in childhood, adolescence and as adults.

**Disclosure:**

No significant relationships.

